# The prevalence of cryptococcal antigen (CrAg) and benefits of pre-emptive antifungal treatment among HIV-infected persons with CD4+ T-cell counts < 200 cells/μL: evidence based on a meta-analysis

**DOI:** 10.1186/s12879-020-05126-z

**Published:** 2020-06-12

**Authors:** Yao Li, Xiaojie Huang, Hui Chen, Yuanyuan Qin, Jianhua Hou, Aixin Li, Hao Wu, Xiaofeng Yan, Yaokai Chen

**Affiliations:** 1 Division of infectious Diseases, Chongqing Public Health Medical Center, 109 Baoyu Road, Shapingba District, Chongqing, 400036 China; 2 Center for Infectious Diseases, Beijing Youan Hospital, Capital Medical University, Beijing, China; 3 School of Biomedical Engineering, Capital Medical University, Beijing, China; 4 Section of Medical Affairs Administration, Chongqing Public Health Medical Center, Chongqing, China

**Keywords:** HIV, Cryptococcal antigenemia, Screening, CD4 + T cell count, Pre-emptive treatment, Meta-analysis

## Abstract

**Background:**

Current WHO guidelines (2018) recommend screening for cryptococcal antigen (CrAg) in HIV-infected persons with CD4+ T cell counts< 100 cells/μL, followed by pre-emptive antifungal therapy among CrAg positive (CrAg+) persons, to prevent cryptococcal meningitis related deaths. This strategy may also be considered for those persons with a CD4+ T cell count of < 200 cells/uL according the WHO guidelines. However, there is sparse evidence in the literature supporting CrAg screening and pre-emptive antifungal therapy in those HIV-infected persons with this CD4+ T cell counts< 200 cells/μL.

**Method:**

We conducted a meta-analysis using data extracted from randomized controlled studies (RCTs) and cohort studies found in a search of Pubmed, Web of Science, the Cochrane Library and the EMBASE/MEDLINE database.

**Results:**

The pooled prevalence of CrAg positivity in HIV-infected persons with CD4+ T cell counts< 200 cells/μL was 5% (95%CI: 2–7). The incidence of CM in CrAg+ persons was 3% (95%CI: 1–6). Among those CrAg+ persons who did not receive pre-emptive treatment, or those who received placebo, the incidence of CM was 5% (95%CI: 2–9), whereas the incidence of CM among those who received pre-emptive antifungal therapy was 3% (95%CI: 1–6), which is a statistically significant reduction in incidence of 40% (RR: 7.64, 95%CI: 2.96–19.73, *p* < 0.00001). As for persons with CD4+ T cell counts between 101 ~ 200 cells/μL, the risk ratio for the incidence of CM among those receiving placebo or no intervention was 1.15, compared to those receiving antifungal treatment (95%CI: 0.16–8.13).

**Conclusions:**

In our meta-analysis the incidence of CM was significantly reduced by pre-emptive antifungal therapy in CrAg+ HIV-infected persons with CD4 <  200 cells/μL. However, more specific observational data in persons with CD4+ T cell counts between 101 ~ 200 cells/μL are required in order to emphasize specific benefit of CrAg screening and pre-emptive antifungal treating in CrAg+ persons with CD4+ T cell counts < 200 cells/μL.

## Background

Cryptococcal meningitis (CM) continues to cause significant mortality in HIV-infected individuals [1, 2], and results in 181,100 deaths globally each year [3]. In resource-limited regions such as sub-Saharan Africa, 15% of HIV-related deaths are due to CM [3]. However, it is possible to detect cryptococcal antigen (CrAg) in blood several weeks to months (22 days on average) before the onset of signs and symptoms of meningitis [4, 5], and thus, the presence of CrAg in blood may be used as a marker for initiation of pre-emptive antifungal therapy in HIV-infected individuals with low CD4+ T cell counts. Previous studies have emphasized that pre-emptive antifungal therapy in CrAg+ persons is imperative to prevent death [6–8]. The prevalence of CrAg positivity among HIV-infected individuals can be considerable, ranging between 1 to 16% in several African and Southeast Asian countries [9], and among persons with CD4+ T cells counts< 100 cells/μL, the prevalence of CrAg positivity averages 7%, with regional variations in prevalence [3]. CrAg positivity resulted in a 20% increase in mortality after antiretroviral therapy (ART) initiation [10] if fluconazole therapy was not initiated prior to ART initiation, and the risk of CM in CrAg+ persons may be as high as 25% during the first year of ART, when fluconazole pre-emptive therapy is not prescribed for these patients [11, 12].

According to the 2018 version of the WHO guidelines, routine CrAg screening and pre-emptive antifungal therapy are recommended in treatment-naive HIV persons with CD4+ T cell counts< 100 cells/μL [13]. The guidelines also state that these strategies may also be considered for HIV-infected persons with CD4+ T cell counts< 200 cells/μL [13]. We therefore conducted a meta-analysis to investigate the prevalence of CrAg positivity in HIV-infected patients, and the benefit of pre-emptive antifungal treatment in HIV-infected persons with CD4+ T cell counts< 200 cells/μL.

## Method

### Search strategy and article screening

We searched relevant English articles in Pubmed, Cochrane Library, MEDLINE/EMBASE and Web of Science from inception until the end of March 20th 2020. The search terms we used were as follows: “acquired immunodeficiency syndrome”, “HIV”, “AIDS”, “cryptococcosis”, and “prophylaxis”. We combined these terms by using “and” or “or”. To avoid missing significant articles, we also screened references of previous meta-analyses and their included studies for eligibility.

Two reviewers (Y L, Y Q) independently screened all obtained articles by titles and abstracts. After removing ineligible articles by referring to our inclusion and exclusion criteria, the remaining articles were further selected for full-text reviewing.

### Inclusion and exclusion criteria

#### Inclusion criteria

1. Randomized-controlled studies (RCTs) or cohort studies,
2. Study subjects had baseline CD4+ T cell counts< 200 cells/μL.
3. CrAg serology was tested for study subjects.
4. Fluconazole or other azole medications were used as the intervention

#### Exclusion criteria

We excluded articles if: (1) all of the study subjects were with CD4+ T cell counts< 100 cells/μL; (2) all of the study subjects were diagnosed with CM or asymptomatic CM; (3) sample size was less than 50; or (4) the incidence of CM and all-cause mortality was unreported.

### Data extraction and quality assessment

The data we extracted included first author, publication year, type of study, study duration, study location, total number of study subjects, baseline CD4+ T-cell counts, age, CrAg screening methods, diagnostic methods for CM, CM events, death events, adverse drug effects, and other opportunistic infections. The JBI (Joanna Briggs Institute) Critical Appraisal Checklist for Cohort Studies was used as a quality assessment tool for cohort studies [14]. The potential bias risk of RCTs was assessed using the Cochrane “risk of bias” tool [15].

### Data analysis

Statistical analysis of data related to proportion of CrAg positivity, the incidence of CM, and all-cause mortality were performed by STATA 14 (Statacorp, Texas, USA) with a 95% confidence interval (95%CI). We used random-effects or fixed-effects models in Review manager 5.3 (The Nordic Cochrane Center, Copenhagen) to compare the incidence of CM and all-cause mortality in CrAg+ persons.

We evaluated statistical heterogeneity through visual inspection of forest plots. Statistical heterogeneity was also assessed using *I*^2^ statistics [16], which was considered non-negligible if *I*^2^ > 50%. Herein, random-model was applied if *I*^2^ > 50% and fixed-model was used when *I*^2^ < 50% [17]. Reporting bias was assessed by examining the asymmetry of funnel plots [16].

The study was registered at the International Prospective Register of Systematic Reviews (PROSPERO), and the registration number is CRD42018110980.

## Results

**Fig. 1:**
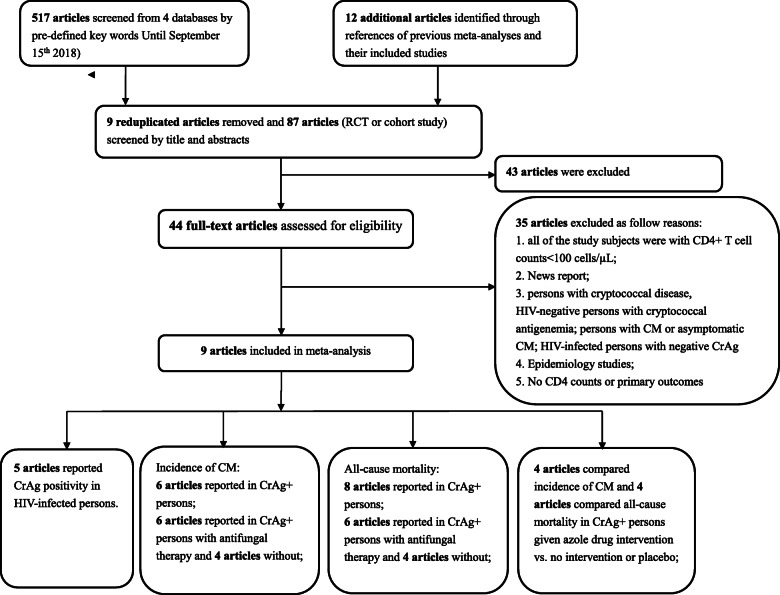
Flow chart of the study selection process

In total, 517 articles were obtained from 4 databases, among which 295 were from Pubmed, 111 were from Web of Science, 13 were from Cochrane Library, and 98 were from MEDLINE/EMBASE. Eighty-four of the 517 articles were RCTs or cohort studies. Additional 12 articles (RCTs or cohort studies) were extracted from references of previous meta-analyses and their included studies, as shown in Fig. 1.

All the 96 RCTs or cohort studies were included for screening. Initially, nine articles (six from Web of Science and three from MEDLINE/EMBASE) were found to be duplicated, and were therefore excluded from the 96 articles. After screening titles and abstracts, 43 of the remaining 87 articles were excluded. Subsequently, 35 articles were excluded from the remaining 44 articles after screening the full-text of each study, among which ten articles only included patients with CD4+ T-cell counts≤100 cells/μL, one article was a news report, three articles reported patients with cryptococcal disease, two articles reported HIV-negative patients with cryptococcal antigenemia, four articles reported data from patients with CM or asymptomatic CM, six articles reported data from HIV-infected patients with negative CrAg, one article reported on the epidemiology of cryptococcosis, and eight articles did not report CD4+ T cell counts or primary outcomes. Finally, a total of 9 articles were included in our meta-analysis.

The characteristics of the 9 included studies were shown in Table 1. Our assessment of quality and potential risk bias in these studies indicated that the following factors could contribute to clinical and methodological heterogeneity, including: (1) the confounding factors or subject recruiting or incomplete follow-up in one of the 8 cohort studies, (2) the unclear risk of attrition in the RCT, and (3) the unclear risk of reporting and other bias in the RCT, as shown in Supplementary Table 1 and Supplementary Table 2. The reporting bias presented by funnel plots were shown in Supplementary Figure 2.

**Table 1:** Characteristics of the 9 included studies

Author, year (reference)	Number of participants	Study type	Study duration	Age (years)	CD4 (cells/μL)	CrAg screening methods	CM diagnostic methods	Location	Therapeutic regimens	Primary outcomes	
										Incidence of CM	All-cause mortality
Chariyalertsak, 2002 [18]	129	Prospective study	104 weeks	18 ~ 60	<200	Not report	Fungal culture, a histopathological examination, or buffy coat smear	Thailand	63 for oral itraconazole (200 mg/day) as group 1; 66 for matched placebo as group 2	0 in group 1; 7 in group 2	12 in group 1; 11 in group 2
Manfredi, 1997 [19]	249	Retrospective study	6 years	22 ~ 59	<200	Not report	Specific polysaccharide antigen detection from body fluids	Italy	128 for oral fluconazole (100 mg/d every third week) as group 1; 121 for no antifungal treatment as group 2	2 in group 1; 9 in group 2	12 in group 1; 13 in group 2
Parkes-Ratanshi, 2011 [20]	1519	Prospective study	42 months	Not report	<200	Not report	CrAg titre> 1:8 on two occasions, or a positive CSF CrAg or *Cryptococcus neoformans* grown from blood or CSF culture	Uganda	760 fluconazole 200 mg 3 times per week for minimum 12 weeks as group 1; 759 allocated to placebo as group 2	1 in group 1; 18 in group 2	0 in group 1; 7 in group 2
McKinsey, 1999 [21]	295	Randomized, placebo-Controlled study	Not report	≥13	<150	Not report	Fungal culture	Not report	149 for itraconazole capsules (200 mg/day) as group 1; 146 for matched placebo as 2	1 in group 1; 8 in group 2	32 in group 1; 21 in group 2
Meya, 2010 [22]	584	Prospective study	30 months	≥18	<200	Not report	Not report	Uganda	Fluconazole (200 ~ 400 mg) for 2 ~ 4 weeks	3 in CrAg+ persons and 0 in CrAg- persons	6 in CrAg+ persons and 0 in CrAg- persons
Kapoor, 2015 [23]	72	Retrospective study	15 months	≥18	<200	LFA	Positive CSF India ink	Sub-Saharan Africa	800 mg fluconazole orally for 2 weeks, followed by 400 mg orally for 2 weeks	0 in CrAg+ persons and 1 in CrAg- persons	2 in CrAg+ persons and 8 in CrAg- persons
Govender, 2015^a^ [1]	1079	Retrospective study	19 months	Not report	< 200	LA or the Latex-*Cryptococcus* antigen detection system	CrAg detected in CSF	South Africa	Fluconazole ranging from 400 to 800 mg per day for at least 3 months	unknow in persons with CD4 < 200	unknow in persons with CD4 < 200
Beyene, 2017 [10]	783	Prospective study	18 months	> 14	≤150	LFA	CSF CrAg	Ethiopia	Fluconazole 800 mg/day 2 weeks, followed by 400 mg/day 8 weeks	2 in CrAg+ persons and 0 in CrAg- persons	4 in CrAg+ persons and 0 in CrAg- persons
Borges, 2019 [24]	214	Prospective study	36 months	> 17	< 200	LFA	India ink microscopy on the CSF, CSF CrAg test and fungal culture	Brazil	Fluconazole 900 mg for 2 weeks, 450 mg for 8 to 10 weeks and a subsequent maintenance dose of 150–300 mg	1 in CrAg+ persons with antifungal treatment and 0 in CrAg+ persons without intervention	2 in CrAg+ persons

“No” means “no data”; “Yes” means “data exists”; OIs means: other opportunity infections

*LFA* Lateral flow assay, *LA* Latex Agglutination, *LP* Lumbar puncture

^a^“Govender, 2015” study was included for evaluating the prevalence of CrAg positivity. Only the data in persons with CD4 < 200 was used

**Fig. 2:**
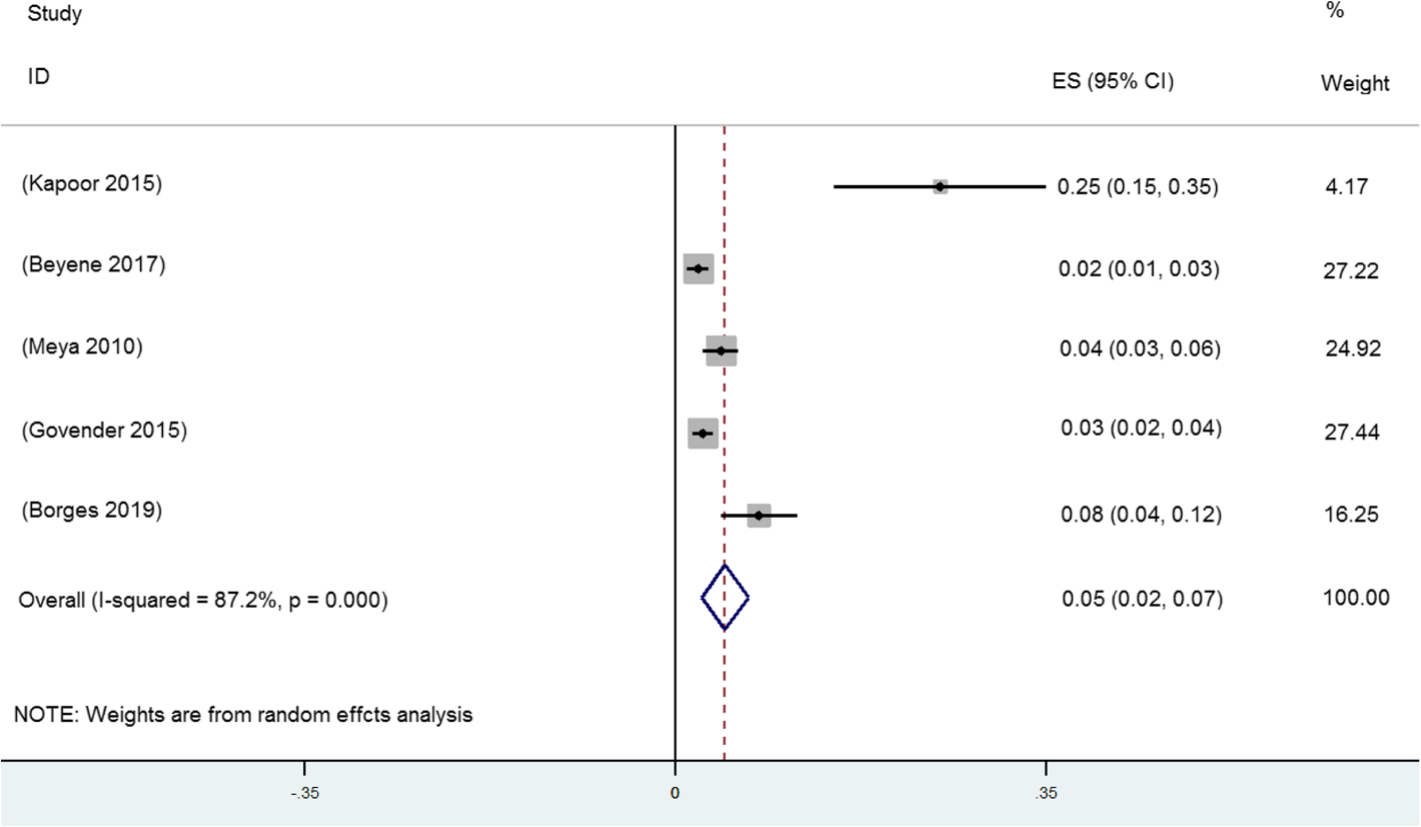
Prevalence of CrAg positivity among HIV-infected persons with CD4+ T cells< 200 cells/μL. Abbreviations: ES = effect size

Five of the 9 included studies reported the prevalence of CrAg positivity (1949 persons with CD4+ T cell counts< 200 cells/μL in four studies; 783 persons with CD4+ T cell counts< 150 cells/μL in one study). The pooled CrAg positivity prevalence in 2732 HIV-infected persons with CD4+ T cell counts < 200 cells/μL was 5% (95%CI: 2–7, *I*^2^ = 87.2%), as shown in Fig. 2.

Six studies reported the incidence of CM among CrAg+ persons (1806 persons with CD4+ T cell counts< 200 cells/μL in four studies; 312 persons with CD4+ T cell counts< 150 cells/μL in two study). The incidence of CM in 2118 CrAg+ persons was 3% (95%CI: 1–5; *P* = 0.021; *I*^2^ = 62.3%), as shown in Table 2 and Supplementary Figure 1a.

**Table 2:** The incidence of CM and all-cause mortality among CrAg+ and CrAg- persons, and among persons with and without antifungal therapy

	Number of reported studies	Number of persons	ES (95%CI)	***I***^**2**^	***P***
**Incidence of CM among CrAg + persons**					
1.1 Incidence of CM among CrAg+ persons	6	2118	0.03 (0.01, 0.05)	62.3%	0.021
**Incidence of CM among persons with and without antifungal therapy**					
2.1 Incidence of CM among persons with antifungal therapy	6	1088	0.03 (0.01, 0.06)	57.7%	0.037
2.2 Incidence of CM among persons without antifungal therapy	4	1092	0.05 (0.02, 0.09)	71.5%	0.015
**All-cause mortality in CrAg+ persons**					
1.1 All-cause mortality among CrAg+ persons	8	2265	0.14 (0.06, 0.22)	94.5%	0.000
**All-cause mortality among persons who with and without antifungal therapy**					
2.1 All-cause mortality among persons with antifungal therapy	6	396	0.17 (0.11, 0.24)	58.3%	0.035
2.2 All-cause mortality among persons without antifungal therapy	4	1092	0.10 (0.01, 0.19)	93.2%	0.000

Six studies reported the incidence of CM among persons who received antifungal therapy (922 persons with CD4+ T cell counts< 200 cells/μL in four studies; 166 persons with CD4+ T cell counts< 150 cells/μL in two studies) and four studies reported the incidence of CM among persons who received placebo or no intervention (946 persons with CD4+ T cell counts< 200 cells/μL in three studies; 146 persons with CD4+ T cell counts< 150 cells/μL in one studies). The incidence of CM of 1088 persons receiving antifungal therapy was 3% (95%CI: 1–6; *P* = 0.037; *I*^2^ = 57.7%), whereas the incidence of CM of 1092 persons in nine studies who received placebo or no intervention was 5%, which equates to a 40% reduction in CM incidence in persons receiving antifungal therapy (95%CI: 2–9; *P* = 0.015; *I*^2^ = 71.5%), as shown in Table 2 and Supplementary Figure 1c and d.

**Fig. 3:**
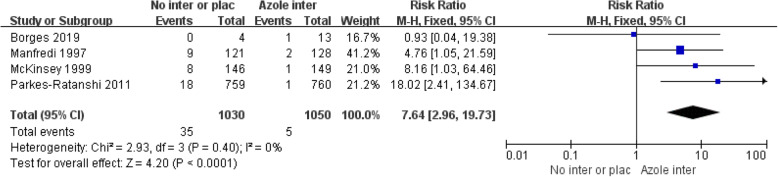
Forest plots of incidence of CM among CrAg + persons receiving azole vs. no intervention or placebo. Abbreviations: M-H, Mantel Haenszel; CI, confidence interval. (“Azole inter” means “Azole drug intervention”, “No inter or plac” mean “No intervention or placebo”)

Four studies compared the incidence of CM between 1030 persons receiving azoles and 1050 persons receiving placebo or no intervention (1785 persons with CD4+ T cell counts< 200 cells/μL in three studies; 295 persons with CD4+ T cell counts< 150 cells/μL in one study). We found that the risk ratio of CM events among persons who received placebo or no intervention was 7.64 times higher than that of those who received antifungal therapy (95%CI: 2.96–19.73; *P* < 0.00001; *I*^2^ = 0%), as shown in Fig. 3.

Eight studies reported all-cause mortality among CrAg+ persons (1953 persons with CD4+ T cell counts< 200 cells/μL in six studies; 312 persons with CD < 150 cells/μL in two studies). The all-cause mortality of 2265 CrAg+ persons was 14% (95%CI: 6–22; *P* = 0.000; *I*^2^ = 93.6%), as shown in Table 2 and Supplementary Figure 1b.

Six studies reported all-cause mortality in persons who received antifungal therapy (230 persons with CD4+ T cell counts< 200 cells/μL in four studies; 166 persons with CD4+ T cell counts< 150 cells/μ in two studies), four studies reported all-cause mortality in persons receiving placebo or no intervention (946 persons with CD4+ T cell counts< 200 cells/μL in three studies; 146 persons with CD4+ T cell counts< 150 cells/μL in one study). The all-cause mortality of 396 persons receiving antifungal therapy was 17% (95%CI: 11–24; *P* = 0.035; *I*^2^ = 58.3%), whereas the all-cause mortality of 1092 CrAg+ persons receiving placebo or no intervention was10%, (95%CI: 1–19; *P* = 0.000; *I*^2^ = 93.2%). Details are shown in Table 2 and Supplementary Figure 1e and f.

**Fig. 4:**
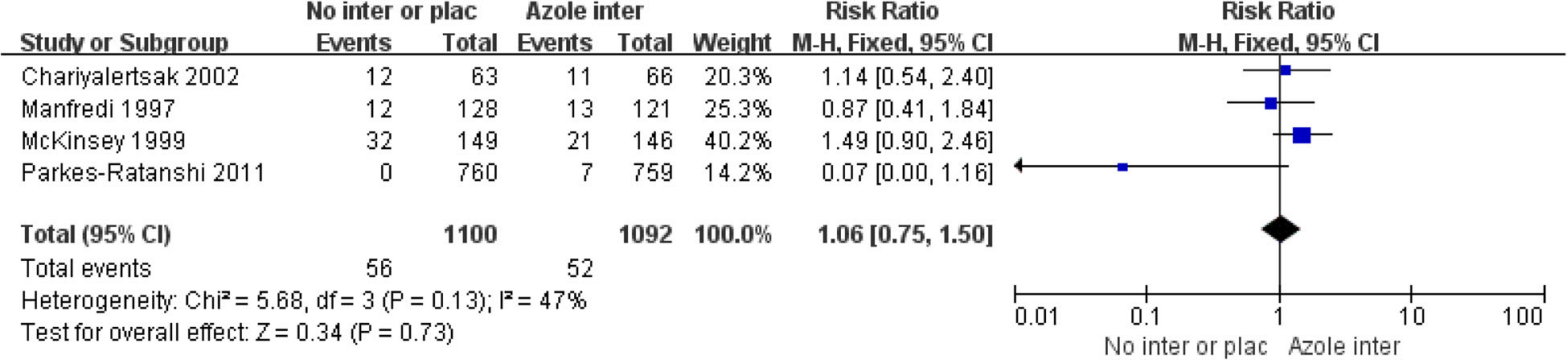
Forest plots of all-cause mortality among CrAg + persons receiving azole vs. no intervention or placebo. Abbreviations: M-H, Mantel Haenszel; CI, confidence interval. (“Azole inter” means “Azole drug intervention”, “No inter or plac” mean “No intervention or placebo”)

Four studies (1897 persons with CD4+ T cell counts< 200 cells/μL in three studies; 295 persons with CD4+ T cell counts< 150 cells/μL in one study) compared all-cause mortality between persons who received azole antifungal therapy and persons who received placebo or no intervention. No significant difference was found in all-cause mortality (risk ratio: 1.06, 95%CI: 0.75–1.50; *P* = 0.73; *I*^2^ = 47%) between 1100 CrAg+ persons who received an azole drug and 1092 CrAg+ persons who received placebo or no intervention (Fig. 4).

In addition, we estimated and compared the prevalence of CrAg positivity, the incidence of CM and all-cause mortality between persons with CD4+ T cell counts< 100 and persons with CD4+ T cell counts between 100 and 200 cells/μL. The results showed that the risk ratio of CrAg positivity prevalence among HIV-infected persons with CD4+ T cell counts< 100 cells/μL was 1.82 times that of those with 100–200 cells/μL (95%CI: 0.77–4.30; *p* = 0.007, *I*^2^ = 63%; three studies; 1886 persons). The risk ratio of the incidence of CM among HIV-infected persons with CD4+ T cell counts< 100 cells/μL was 2.53 times that of those with CD4+ T cell counts between 100 and 200 cells/μL (95%CI: 0.50–12.71, *p* = 0.26, *I*^2^ = 59%; four studies, 1960 persons). The risk ratio of the all-cause mortality among HIV-infected persons with CD4+ T cell counts < 100 cells/μL was 4.15 times that of those with CD4+ T cell counts between 100 and 200 cells/μL (95%CI: 0.89–19.42, *p* = 0.07, I^2^ = 0%; two studies, 1552 persons). Further, the risk ratio of the incidence of CM among persons with CD4+ T cell counts between 100 and 200 cells/μL receiving placebo or no intervention was 1.15 times compared to those receiving antifungal treatment (95%CI: 0.16–8.13, *p* = 0.97, *I*^2^ = 0%; three studies; 140 persons). The risk ratio of the all-cause mortality among persons with CD4+ T cell counts between 100 and 200 cells/μL receiving antifungal treatment was 0.27 compared to those receiving placebo or no intervention (95%CI: 0.01–4.93, *p* and I^2^ not applicable; one study; seven persons). Details are shown in Table 3.

**Table 3:** Comparisons of CrAg positivity, incidence of CM and all-cause mortality among HIV-infected persons stratified by CD4+ T cell count

**Author, year**	**Prevalence of CrAg positivity**		**Incidence of CM**				**All-cause mortality**			
	**CD4<100 (cells/μL)**	**CD4 100 ~ 200 (cells/μL)**	**CD4<100 (cells/μL)**		**CD4 100 ~ 200 (cells/μL)**		**CD4<100 (cells/μL)**		**CD4 100 ~ 200 (cells/μL)**	
			**with antifungal treatment**	**without antifungal treatment**	**with antifungal treatment**	**without antifungal treatment**	**with antifungal treatment**	**without antifungal treatment**	**with antifungal treatment**	**without antifungal treatment**
Chariyalertsak, 2002 [25]	Not reported		0 of 40	6 of 45	0 of 23	0 of 21	Not reported		Not reported	
Parkes-Ratanshi, 2011 [18]	Not reported		17 of 698		2 of 821		3^a^ of 698		0^a^ of 821	
McKinsey, 1999 [19]	Not reported		0 of 101	7 of 103	1 of 48	1 of 43	Not reported		Not reported	
Meya, 2010 [21]	26 of 295	7 of 298	3 of 21	2 of 5	Not reported		6 of 21	5 of 5	0 of 4	1 of 3
Govender, 2015 [1]	20 of 708	6 of 371	Not reported		Not reported		Not reported		Not reported	
Borges, 2019 [24]	12 of 159	5 of 5	0 of 9	0 of 3	0 of 1	1 of 4	Not reported		Not reported	
	**Pooled CrAg + prevalence:** 0.06 [−0.02, 0.11], *p* = 0.001, I^2^ = 86.5%	**Pooled CrAg + prevalence:** 0.02 [0.01, 0.03], *p* = 0.146, I^2^ = 48%	**Risk ratio with 95%CI:** 7.67 [2.03, 29.05], *p* = 0.36, I^2^ = 1%		**Risk ratio with 95%CI:** 1.15 [0.16, 8.13], *p* = 0.97, I^2^ = 0%		**Risk ratio with 95%CI:** 0.32 [0.16, 0.64], *p* and I^2^ not applicable		**Risk ratio with 95%CI:** 0.27 [0.01, 4.93], *p* and I^2^ not applicable	
**Risk ratio with 95%CI**	1.82 [0.77, 4.30], *p* = 0.007, *I*^2^ = 63%		2.53 [0.50, 12.71], *p* = 0.26, *I*^2^ = 59%				4.15 [0.89, 19.42], *p* = 0.07, *I*^2^ = 0%			

^a^ Died within 4 weeks

## Discussion

Several meta-analyses have been conducted in the past designed to evaluate the necessity of CrAg screening and administration of pre-emptive antifungal treatment among HIV-infected CrAg+ persons with varying low CD4 levels. For example, Temfack et al investigated the effectiveness of CrAg detection and the initiation of pre-emptive fluconazole treatment in HIV-infected persons with cryptococcal antigenemia and CD4+ T cell levels< 100 cells/μL [16]. Their results suggested that administration of fluconazole pre-emptive therapy to CrAg+ persons greatly reduced the risk of incident CM, and may have specific survival benefits [16]. Another meta-analysis conducted by Ssekitoleko et al also suggested that in resource-limited settings, CrAg+ persons should routinely receive primary antifungal prophylaxis [25], but they failed to clarify at which specific CD4+ T cell count antifungal prophylaxis should be initiated. Ford et al’s [26] meta-analysis only reported the combined prevalence of cryptococcal antigenemia among HIV-infected persons with CD4+ T cell counts≤100 cells/μL, and with CD4+ T cell counts between 101 ~ 200 cells/μL. Importantly, their study did not mention whether pre-emptive antifungal treatment was necessary or effective among HIV-infected persons with cryptococcal antigenemia at these two CD4+ T cell count strata. From the above studies, it may be gathered that the prudence and benefits of CrAg screening and pre-emptive antifungal therapy remain unclear at higher CD4+ T cell counts. The objective of our meta-analysis was to investigate the prevalence of cryptococcal antigenemia in HIV-infected patients with CD4+ T cell counts< 200 cells/μL, and the potential benefit of pre-emptive antifungal therapy among HIV-infected persons with cryptococcal antigenemia and CD4+ T cell counts< 200 cells/μL.

The pooled prevalence of CrAg positivity in HIV-infected persons with CD4+ T cell counts< 200cells/μL was 5% (5 studies) in our meta-analysis, which was similar to 6% (31 studies) among HIV-infected persons with CD4+ T cell counts< 100cells/μL in Temfack’s meta-analysis [16] and 6.5% (60 studies) among HIV-infected persons with CD4+ T cell counts < 100 cells/μL in Ford’s meta-analysis [26]. Therefore, antifungal prophylaxis seems imperative for HIV-infected persons with cryptococcal antigenemia who have CD4+ T cell counts< 200 cells/μL.

Our results have demonstrated that, in persons with CD4+ T cell count< 200 cells/μL, the risk ratio of CM events among those who received placebo or no intervention was significant higher than those who received antifungal therapy, suggesting that antifungal prophylaxis significantly reduce the risk of CM events in CrAg+ persons with a higher CD4+ T-cell counts. However, the very limited data among persons with CD4+ T cell counts between 101 ~ 200 cells/μL restricted our capacity to investigate it further. Thus, more specific data are needed to demonstrate the benefit of antifungal treatment in HIV-infected persons with CD4+ T cell counts between 100 and 200 cells/μL, and warrants further investigation.

No significant difference in all-cause mortality was found in our meta-analysis among CrAg+ persons who received pre-emptive antifungal therapy versus placebo or no intervention. This is a somewhat surprising outcome, and the reason of this may be associated with the discrepant sample sizes in these two groups (396 vs. 1092).

We considered the following possible reasons for clinical and methodological heterogeneity: discrepancies in follow-up time for reporting CM events and death events, variations in drug dosing, regimens, or drug class of prescribed antifungal therapy, ART status of subjects, and risk of bias. For example, the study durations ranged from104 weeks to 6 years, and the dosing of azole antifungal treatments ranged from 100 mg/d to 900 mg/d. With regards to reporting bias, it is possible that the unformed funnel plot for all-cause mortality could be a consequence of the varied ART status of study participants, different dosage regimens and duration of treatment and the different follow-up periods in each of the individual studies.

There are some limitations in our study. Firstly, the data supporting the association between prevalence of CrAg positivity and occurrence of adverse outcomes in HIV-infected persons with CD4+ T-cell counts between 100 and 200 cells cells/μL is sparse. Secondly, there exists a paucity of new data regarding CrAg positivity prevalence, CM incidence, and all-cause mortality in HIV-infected persons with CD4+ T-cell counts< 200 cells/μL since 2015 [27], and our pooled outcome analyses relied heavily on older studies, which may be less applicable to the modern test-and-treat era. And thirdly, the dosage and durations of azole therapy was not assessed in our meta-analysis. The preceding limitations may contribute to the clinical and methodological heterogeneity in our study.

## Conclusions

In our meta-analysis, the incidence of CM was significantly reduced by pre-emptive antifungal therapy in CrAg+ persons with CD4+ T cell counts< 200 cells/μL. Nevertheless, more specific intervention data are needed in persons with CD4+ T cell counts between 101 ~ 200 cells/μL to better clarify the benefit of CrAg screening and pre-emptive antifungal treating in CrAg- persons with CD4+ T cell counts< 200 cells/μL more clear.

## Supplementary information

**Additional file 1: Figure S1.** Incidence of CM and all-cause mortality among CrAg+ persons with and without antifungal therapy, (a) Incidence of CM among CrAg+ persons; (b) all-cause mortality among CrAg+ persons; (c) Incidence of CM among persons with antifungal therapy and without antifungal therapy (d); (e) all-cause mortality among persons with antifungal therapy and without antifungal therapy (f). **Figure S2.** Funnel plots. Funnel plots of the incidence of CM and all-cause mortality among patients with CD4 <  200 cells/μL. **Table S1.** Quality assessment of 8 included studies by using the JBI Critical Appraisal Checklist for Cohort Studies. **Table S2.** Risk of bias of the 1 included RCT.

## Data Availability

All the data and materials are available from Pubmed, Cochrane Library, MEDLINE/EMBASE and Web of Science.

## Abbreviations

CrAg: Cryptococcal antigen
HIV: Human immunodeficiency virus
CrAg+: CrAg positive
RCTs: Randomized controlled studies
RR: Risk ratio
95%CI: Confidence interval
CrAg-: CrAg negative
CM: Cryptococcal meningitis
ART: Antiretroviral therapy
WHO: World Health Organization
PROSPERO: Prospective Register of Systematic Reviews (PROSPERO)
