# Correction to: The prevalence of cryptococcal antigen (CrAg) and benefits of pre-emptive antifungal treatment among HIV-infected persons with CD4+ T-cell counts < 200 cells/μL: evidence based on a meta-analysis

**DOI:** 10.1186/s12879-021-06132-5

**Published:** 2021-06-24

**Authors:** Yao Li, Xiaojie Huang, Hui Chen, Yuanyuan Qin, Jianhua Hou, Aixin Li, Hao Wu, Xiaofeng Yan, Yaokai Chen

**Affiliations:** 1 Division of infectious Diseases, Chongqing Public Health Medical Center, 109 Baoyu Road, Shapingba District, Chongqing, 400036 China; 2 Center for Infectious Diseases, Beijing Youan Hospital, Capital Medical University, Beijing, China; 3 School of Biomedical Engineering, Capital Medical University, Beijing, China; 4 Section of Medical Affairs Administration, Chongqing Public Health Medical Center, Chongqing, China

**Correction to: BMC Infect Dis 20, 410 (2020)**

**https://doi.org/10.1186/s12879-020-05126-z**

Following publication of the original article [1], the authors identified an error in Fig. 3. The correct figure is given below.

**Fig. 3:**
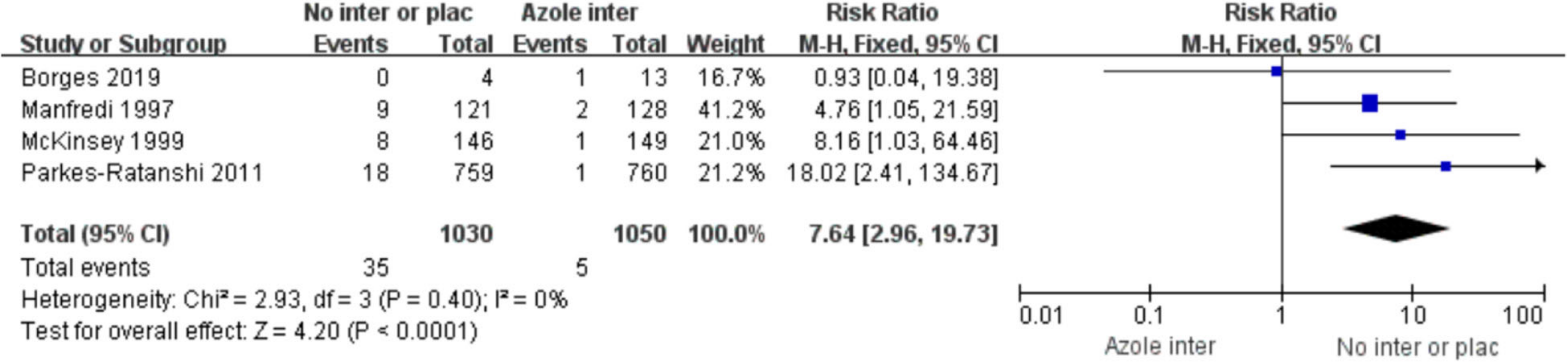
Forest plots of incidence of CM among CrAg + persons receiving azole vs. no intervention or placebo. Abbreviations: M-H, Mantel Haenszel; CI, confidence interval. (“Azole inter” means “Azole drug intervention”, “No inter or plac” mean “No intervention or placebo”

The original article [1] has been corrected.
