# Supplementary material for: The prevalence of cryptococcal antigen (CrAg) and benefits of pre-emptive antifungal treatment among HIV-infected persons with CD4+ T-cell counts < 200 cells/μL: evidence based on a meta-analysis
Source: BMC Infect Dis. 2020 Jun 12;20:410. doi: 10.1186/s12879-020-05126-z (PMC7291520; doi:10.1186/s12879-020-05126-z)
Supplement: Supplementary file 1 — Additional file 1: Figure S1. Incidence of CM and all-cause mortality among CrAg+ persons with and without antifungal therapy, (a) Incidence of CM among CrAg+ persons; (b) all-cause mortality among CrAg+ persons; (c) Incidence of CM among persons with antifungal therapy and without antifungal therapy (d); (e) all-cause mortality among persons with antifungal therapy and without antifungal therapy (f). Figure S2. Funnel plots. Funnel plots of the incidence of CM and all-cause mortality among patients with CD4 < 200 cells/μL. Table S1. Quality assessment of 8 included studies by using the JBI Critical Appraisal Checklist for Cohort Studies. Table S2. Risk of bias of the 1 included RCT. [file 12879_2020_5126_MOESM1_ESM.doc]

**Figure S1. Incidence of CM and all-cause mortality among CrAg+ persons with and without antifungal therapy, (a)** Incidence of CM among CrAg+ persons; (b) all-cause mortality among CrAg+ persons; (c) Incidence of CM among persons with antifungal therapy and without antifungal therapy (d); (e) all-cause mortality among persons with antifungal therapy and without antifungal therapy (f).

**Figure S2. Funnel plots.** Funnel plots of the incidence of CM and all-cause mortality among patients with CD4<200 cells/µL.

**Table S1.** Quality assessment of 8 included studies by using the JBI Critical Appraisal Checklist for Cohort Studies.

**Table S2.** Risk of bias of the 1 included RCT.

| **a** | **Incidence of CM among CrAg+ persons** | **b** | **All cause mortality among CrAg+ persons** |
| --- | --- | --- | --- |
|  | **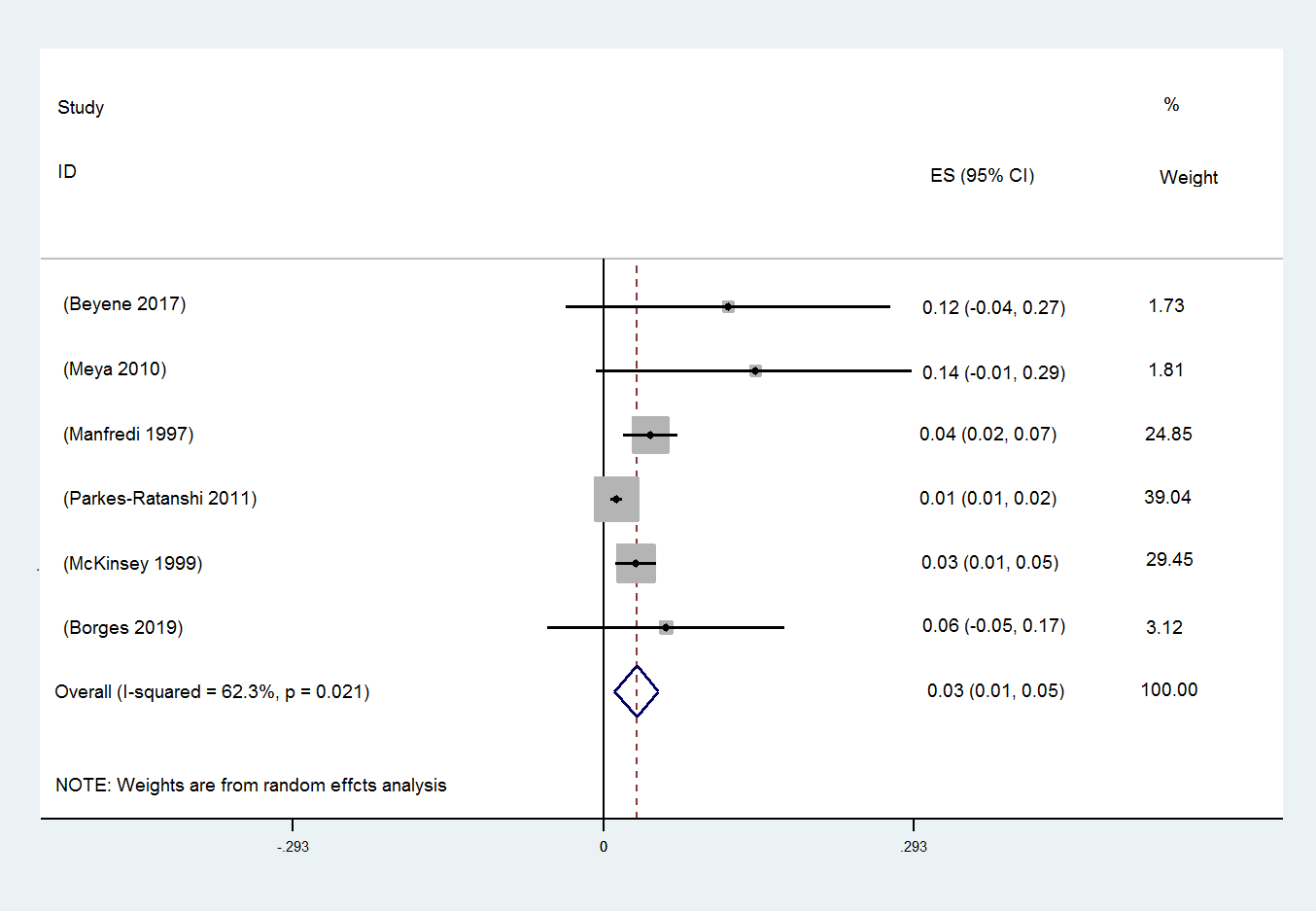** |  | **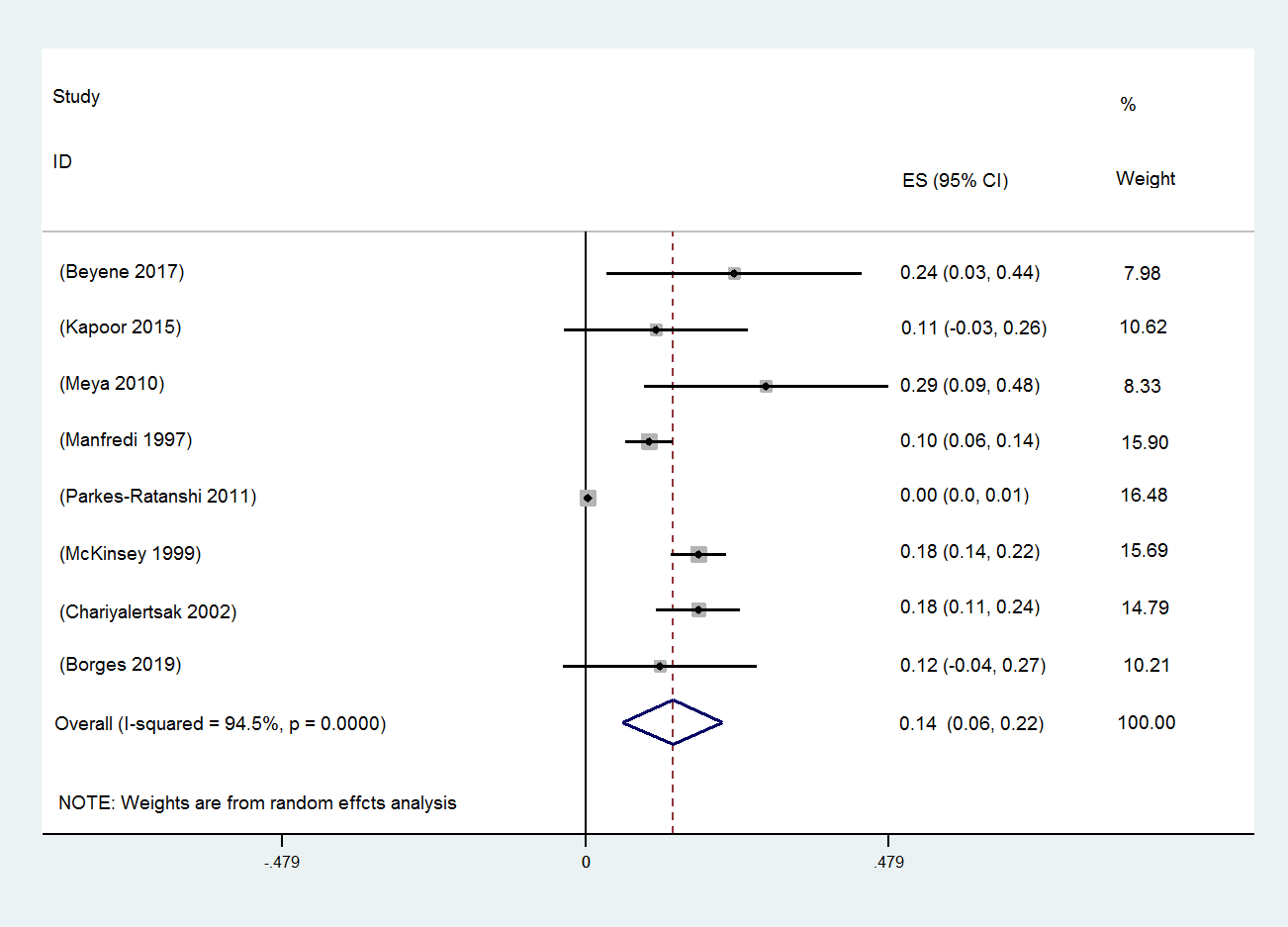** |
| **c** | **Incidence of CM among persons with antifungal therapy** | **d** | 1. **Incidence of CM among persons without antifungal therapy** |
|  | **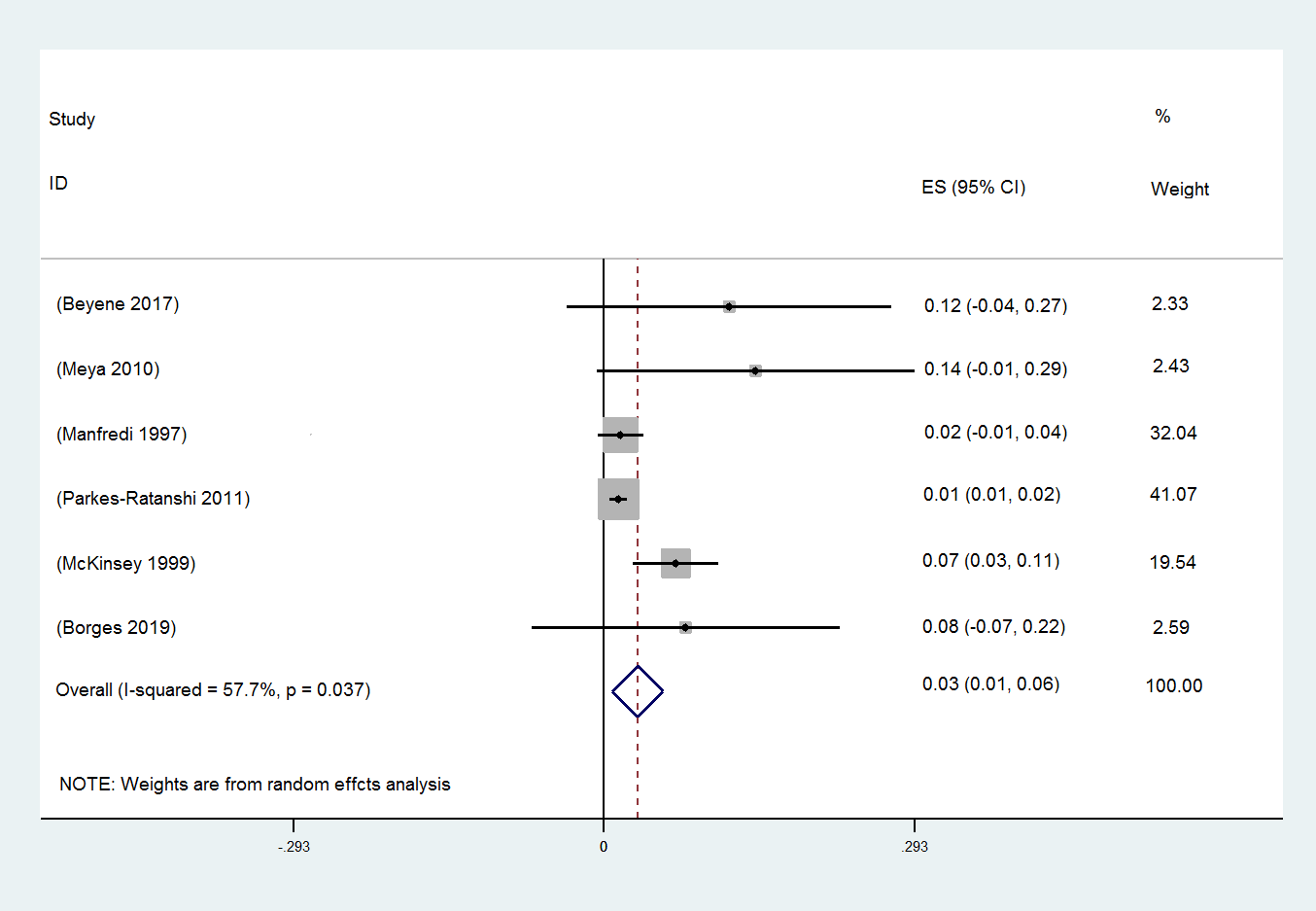** |  | **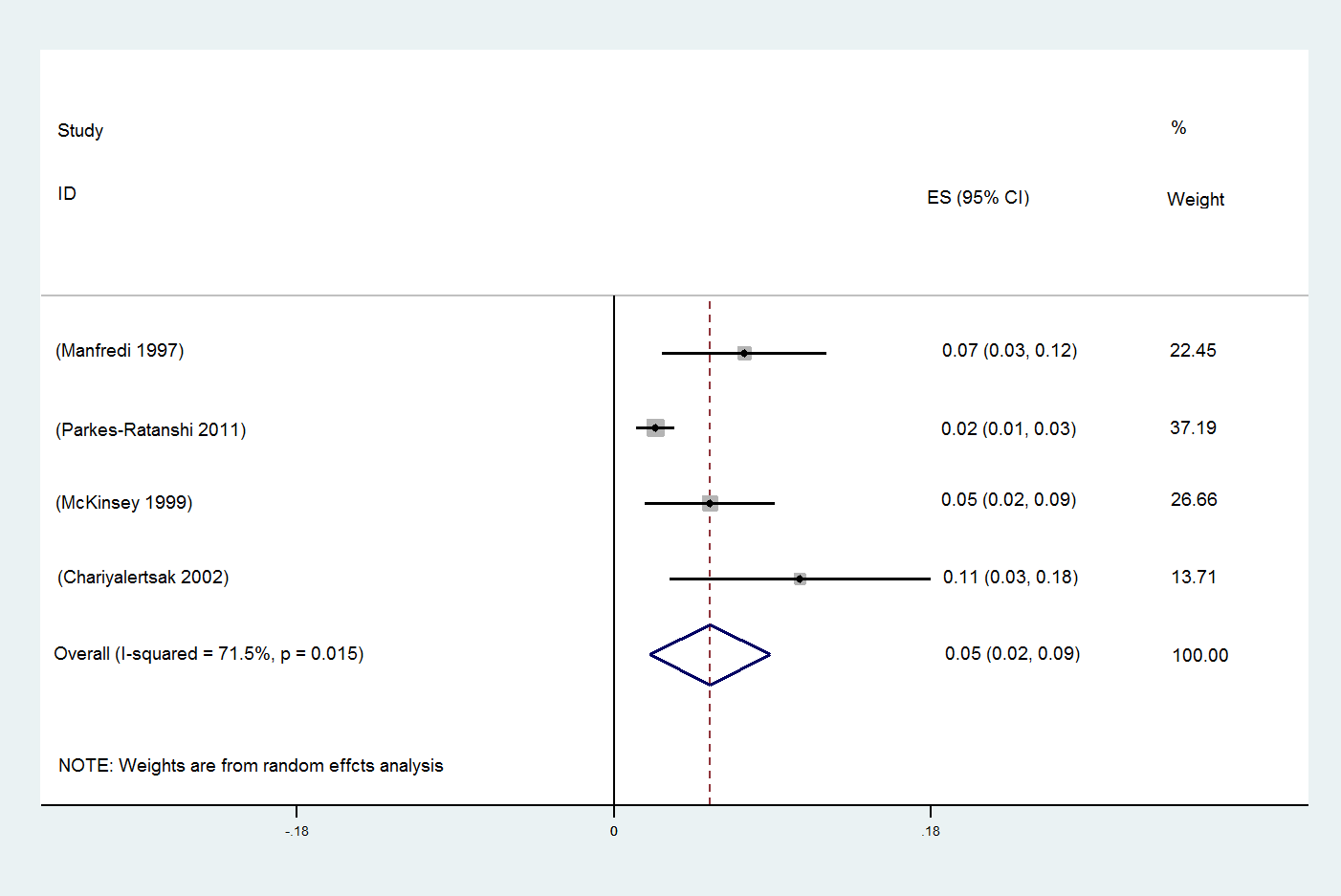** |
| **e** | **All cause mortality among persons with antifungal therapy** | **f** | **All cause mortality among persons without antifungal therapy** |
|  | **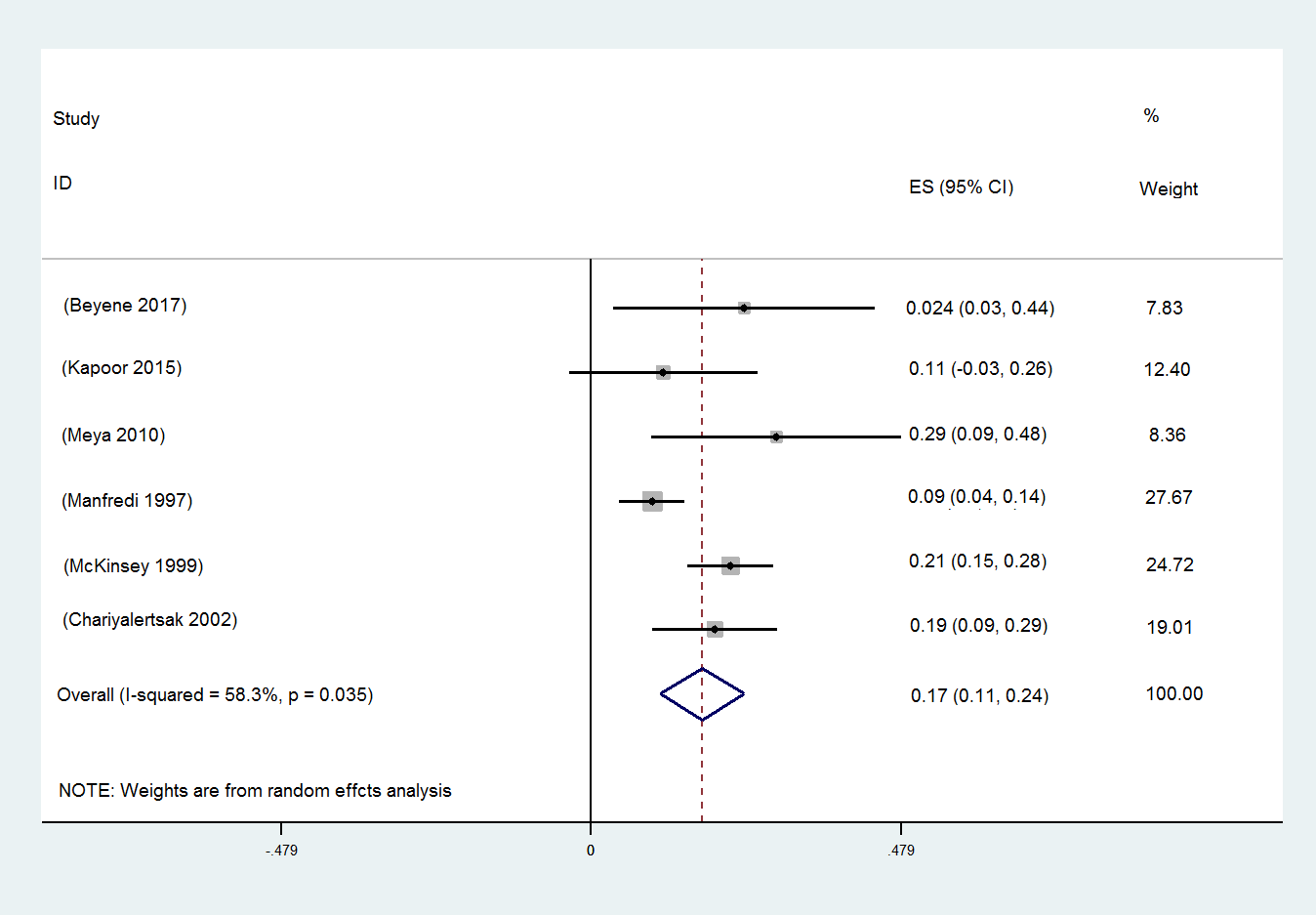** |  | **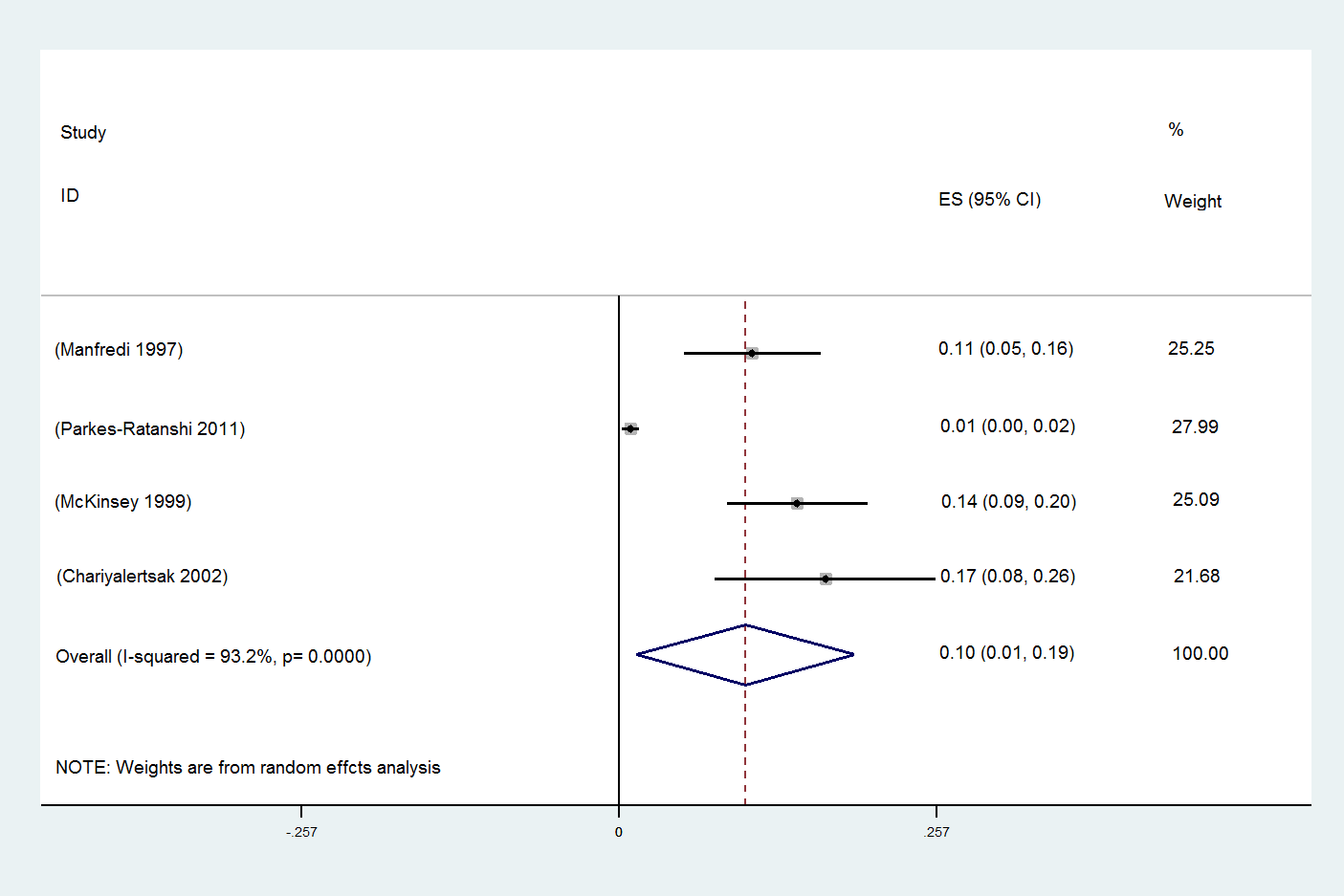** |

**Figure S1. Incidence of CM and all-cause mortality among CrAg+ persons with and without antifungal therapy, (a)** Incidence of CM among CrAg+ persons; (b) all-cause mortality among CrAg+ persons; (c) Incidence of CM among persons with antifungal therapy and without antifungal therapy (d); (e) all-cause mortality among persons with antifungal therapy and without antifungal therapy (f).

| 1. Incidence of CM among CrAg+ patients receiving azole vs. no intervention or placebo | 2. All cause mortality among CrAg+ patients receiving azole vs. no intervention or placebo |
| --- | --- |
| 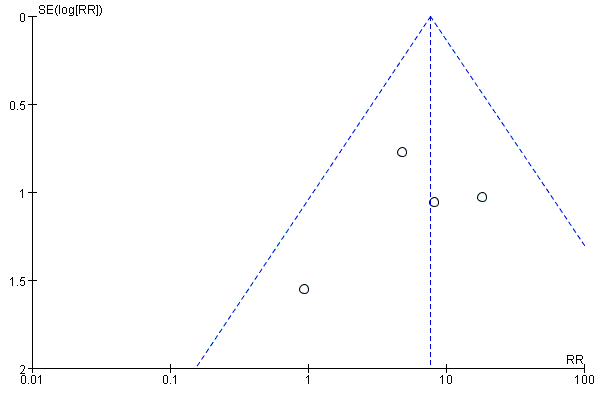 | 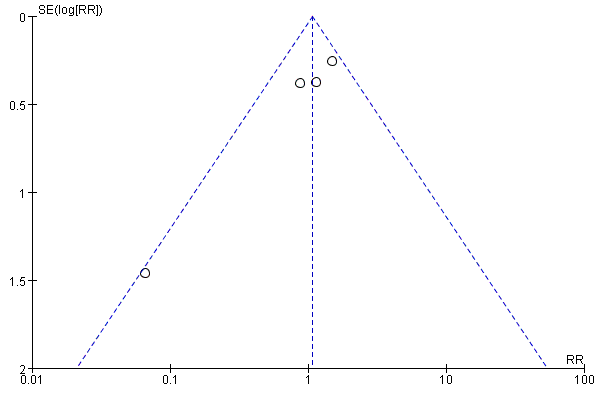 |

**Figure S2. Funnel plots.** Funnel plots of the incidence of CM and all-cause mortality among CrAg+ patients receiving azole vs. no intervention or placebo.

**Table 1.** Quality assessment of 8 included cohort studies by using the JBI Critical Appraisal Checklist for Cohort Studies.

|  | Manfredi, 1997 | Parkes-  Ratanshi,  2011 | Kapoor,  2015 | Beyene,  2017 | Meya,  2010 | Govender  2015 | Chariyalertsak, 2002 | Borges, 2019 |
| --- | --- | --- | --- | --- | --- | --- | --- | --- |
| 1. Were the two groups similar and recruited from the same population? | Yes | Yes | Not applicable | Not applicable | Not applicable | No | Yes | Yes |
| 1. Were the exposures measured similarly to assign people to both exposed and unexposed groups? | Yes | Yes | Not applicable | Not applicable | Not applicable | Not applicable | Yes | Yes |
| 1. Was the exposure measured in a valid and reliable way? | Yes | Yes | Yes | Yes | Yes | Yes | Yes | Yes |
| 1. Were confounding factors identified? | No | Unclear | Unclear | Yes | Yes | Yes | Yes | Unclear |
| 1. Were strategies to deal with confounding factors stated? | No | Unclear | Unclear | Unclear | Yes | Yes | Yes | Unclear |
| 1. Were the goups/participants free of the outcome at the start of the study (or at the moment of exposure)? | Yes | Yes | Yes | Yes | Yes | Yes | Yes | Yes |
| 1. Were the outcomes measured in a valid and reliable way? | Yes | Yes | Yes | Yes | Yes | Yes | Yes | Yes |
| 1. Was the follow up time reported and sufficient to be long enough for outcomes to occur? | Yes | Yes | Yes | Yes | Yes | Yes | Yes | Yes |
| 1. Was follow up complete, and if not, were the reasons to loss to follow up described and explored? | Yes | Yes | Unclear | Yes | Yes | Yes | No | Yes |
| 1. Were strategies to address incomplete follow up utilized? | Unclear | Unclear | Unclear | Unclear | Unclear | Unclear | Uclear | Unclear |
| 1. Was appropriate statistical analysis used? | Yes | Yes | Yes | Yes | Yes | Yes | Yes | Yes |

**Table S2.** Risk of bias of the 1 included RCT.

| **Study** | **Bias** | **Authors’ judgment** | **Supporting evidence** |
| --- | --- | --- | --- |
| McKinsey,  1999 | Random sequence generation (selection bias) | Low risk | Randomization was stratified by site, and each site had an independent randomization code |
|  | Allocation concealment (selection bias) | Low risk | “Subjects were randomized in a 1:1 ratio to take either two 100-mg itraconazole capsules once daily or two placebo capsules, which were identical in appearance to the itraconazole capsules ” |
|  | Blinding of participants and researches (performance bias) | Low risk | “double-blind Study” |
|  | Blinding of outcome assessment (detection bias) | Low risk | “double-blind Study” |
|  | Incomplete outcome data (attrition bias) | Unclear risk | No description |
|  | Selective reporting (reporting bias) | Unclear risk | No description |
|  | Other bias | Unclear risk | No description |
